# α-synuclein-lanthanide metal ions interaction: binding sites, conformation and fibrillation

**DOI:** 10.1186/s13628-016-0026-1

**Published:** 2016-02-03

**Authors:** Jia Bai, Zeting Zhang, Maili Liu, Conggang Li

**Affiliations:** 1 Key Laboratory of Magnetic Resonance in Biological Systems, State Key Laboratory of Magnetic Resonance and Atomic and Molecular Physics, National Center of Magnetic Resonance in Wuhan, Wuhan Institute of Physics and Mathematics, Chinese Academy of Sciences, Wuhan, 430071 P.R. China; 2 University of Chinese Academy of Sciences, Beijing, 100049 China

**Keywords:** α-synuclein (αS), Lanthanide metal ions, Binding sites, Fibrillation

## Abstract

**Background:**

The pathological hallmark of Parkinson’s disease is the deposition of cytoplasmic neuronal inclusions termed Lewy bodies. The major component of Lewy bodies is amyloid fibrils of α-synuclein. To investigate what causes α-synuclein aggregation is essential to understand its pathological roles in Parkinson’s disease. Various metal ions, including iron and copper, have been implicated in the pathogenesis of Parkinson’s disease. Divalent metal ions can regulate α-synuclein fibrillation rate, however, few studies have been performed to investigate how trivalent metal ions interact with α-synuclein and their effect on α-synuclein fibrillation. The study of the interaction between divalent and trivalent metal ions with α-synuclein is of vital importance to realize the mechanism of α-synuclein fibrillation.

**Results:**

Here we used nuclear magnetic resonance spectroscopy to determine the trivalent metal ions (lanthanides) binding sites in α-synuclein. We found that lanthanide metal ions not only bind non-specifically to the C-terminal domain of α-synuclein, but also transiently interact with residues contain carboxyl groups in the N-terminal and NAC regions, the latter binding sites were not found for divalent cations. In addition, lanthanide ions bound α-synuclein exhibits slower conformational exchange rate. Compare to divalent cations, lanthanide ions accelerate α-synuclein fibrillation much faster.

**Conclusions:**

We identified the lanthanide metal ions binding sites in α-synuclein and found a hierarchal effect for lanthanide ions binding to α-synuclein, driven by the interaction with aspartic acids and glutamic acids residues. Lanthanide ions binding also induced conformational dynamics change of α-synuclein. Compared to divalent cations, lanthanide metal ions significantly accelerated α-synuclein fibrillation, possibly due to the different inherent properties such as charge, binding sites and coordination modes.

**Electronic supplementary material:**

The online version of this article (doi:10.1186/s13628-016-0026-1) contains supplementary material, which is available to authorized users.

## Background

Parkinson's disease (PD) is a common neurodegenerative disease of the population over 65 [1]. The histological hallmark of Parkinson’s disease is the selective missing of dopaminergic neurons in the substantia nigra pars compacta. Intraneuronal deposits of fibrillar and misfolded proteins called the Lewy bodies appear in the affected brain areas. The main component of the Lewy bodies is α-synuclein (αS) aggregates [1–3]. Investigating what causes αS aggregation is important to understand its pathological roles in PD.

In aqueous solution, monomeric αS is a natively unfolded protein with no apparent ordered secondary structure detectable by Far-UV Circular Dichroism (CD), Fourier transform infrared spectroscopy, or nuclear magnetic resonance (NMR) spectroscopy [4–6], but it forms β-sheet riched amyloid like fibrils under certain conditions [4, 7–9]. The N-terminal of αS exhibits a partially α-helical secondary structure upon binding with negatively phospholipid membranes and detergent micelles, while the C-terminus still remains dynamically unstructured [10–14]. αS has 140 amino acids with three distinct regions: the positively charged N-terminal region (residues 1–60); the hydrophobic NAC (non-amyloid β component) region (residues 61–95); and the highly negatively charged C-terminal region (residues 96–140) [15, 16]. The exposure of NAC region is considered as the main reason for αS fibrillation [16]. αS has the structural properties of auto-inhibition fibrillation due to the long-range transient intra-molecular interaction between the N-terminus and the C-terminus, which protecting NAC region from exposure [17–20].

Increasing evidences have shown that altered metal homeostasis might be involved in the progression of neurodegenerative diseases. The possible involvement of heavy metals in the etiology of PD followed primarily from the results of epidemiological studies [21–24] and Lewy bodies component analysis in the parkinsonian substantia nigra [25, 26]. The potential link between metal ions and the PD related protein αS was observed in in vitro experiments. Recent studies show that the binding affinities of αS for diverse metal ions are different, but the binding sites are similar for the majority of metal ions. For divalent cations such as Fe^2+^, Mn^2+^, Co^2+^,Ni^2+^ and Ca^2+^, they all bound non-specifically to the C-terminal domain of αS [27–29]. But for Cu^2+^, it has high affinity to the αS N-terminus, and low affinity to the C-terminus [27, 30, 31]. No conformational change of αS was observed at low ionic concentration of various ions. At high concentration of metal ions, K^+^, Na^+^, Li^+^, Cs^+^, and Ca^2+^ has no effect on the unfolded structure of αS, but Mn^2+^, Cd^2+^, Mg^2+^ and Zn^2+^ induce a small increase in α-helix contents, and Cu^2+^, Co^2+^, Fe^3+^ and Al^3+^ induce more α-helix contents [32, 33].

Recently, studies have shown that lanthanide ions might affect the neuronal systems, but the toxicological behaviours were very complicated and the effects depended on a variety of factors [34–36]. With the increasing applications of lanthanides in industry, agriculture and medicine [37–41], particularly for the people who has long-term exposure in the electronics components industry or mining, public concern pays more and more attention on the toxicity of the lanthanides. In this study, we characterized the interaction between lanthanide metal ions (Ln^3+^) with recombinant human αS and the binding sites (region) were determined using NMR spectroscopy. We found that lanthanide metal ions accelerated αS fibrillation much faster than divalent cations in vitro. Based on the interaction information, we proposed the mechanism by which lanthanide metal ions accelerated αS fibrillation in vitro.

## Results and discussion

### Lanthanide metal ions binding sites determination

**Fig. 1:**
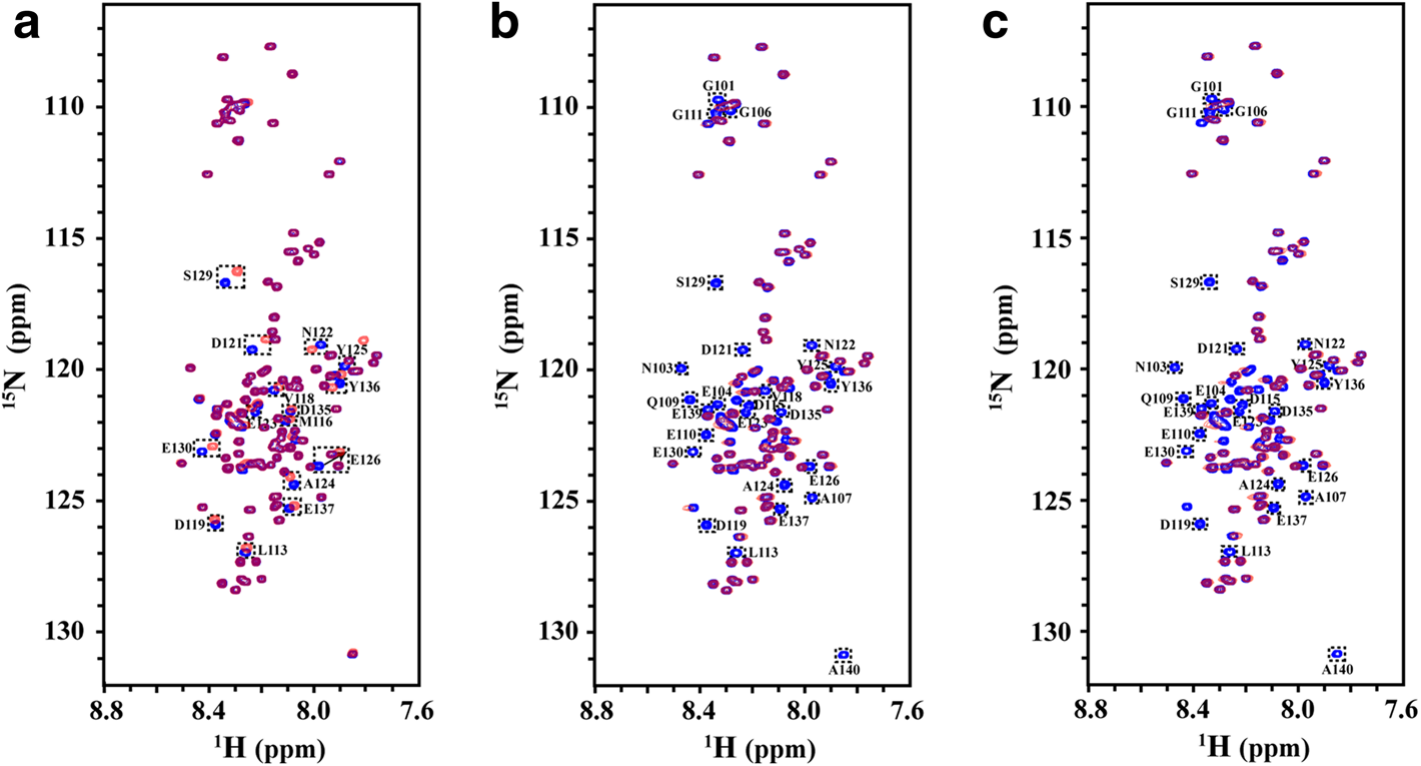
^1^H-^15^N HSQC spectra of α-synuclein in the absence (*blue*) and presence of (*red*) (**a**) Lu^3+^, (**b**) Tb^3+^, (**c**) Dy^3+^ at molar ratios of αS/Ln^3+^(1/1), respectively. All spectra were obtained at 15 °C. Residues with chemical shift change or severely line broadening were labelled in the figure

The ^1^H-^15^N heteronuclear single quantum correlation (HSQC) spectrum that contains one cross peak for each amide group (except proline) in a protein is routinely used to characterize protein-ligand and protein-protein interactions. The lanthanide metal ions (Ln^3+^) binding sites in αS were identified by using chemical shift perturbation of ^1^H-^15^N HSQC NMR spectroscopy of αS in the absence and presence of Ln^3+^ at various concentration ratios. We collected a series of ^1^H-^15^N HSQC spectra of αS from 0 to 1/4 molar ratio of αS/Ln^3+^. Fig. 1 showed ^1^H-^15^N HSQC spectra of αS in the absence (blue) and presence (red) of Ln^3+^ at 1/1 molar ratio of αS/Ln^3+^, respectively. In the presence of diamagnetic Lu^3+^, the residues with significant chemical shift changes were labelled in Fig. 1a, and the assignments were according to Biological Magnetic Resonance Data Bank (BMRB entry number is 16,543 for α-synuclein). In the presence of paramagnetic Tb^3+^ and Dy^3+^, the cross peaks of some residues became severely broadening and disappeared(Fig. 1b and c)due to paramagnetic relaxation enhancement effects, these residues were similar as those showing chemical shift changes in Fig. 1a. The ^1^H -^15^N spectra of αS in the presence of Ln^3+^ were also characteristic spectra of unfolded protein, suggesting that binding with Ln^3+^ might not cause significant structural change of αS.

**Fig. 2:**
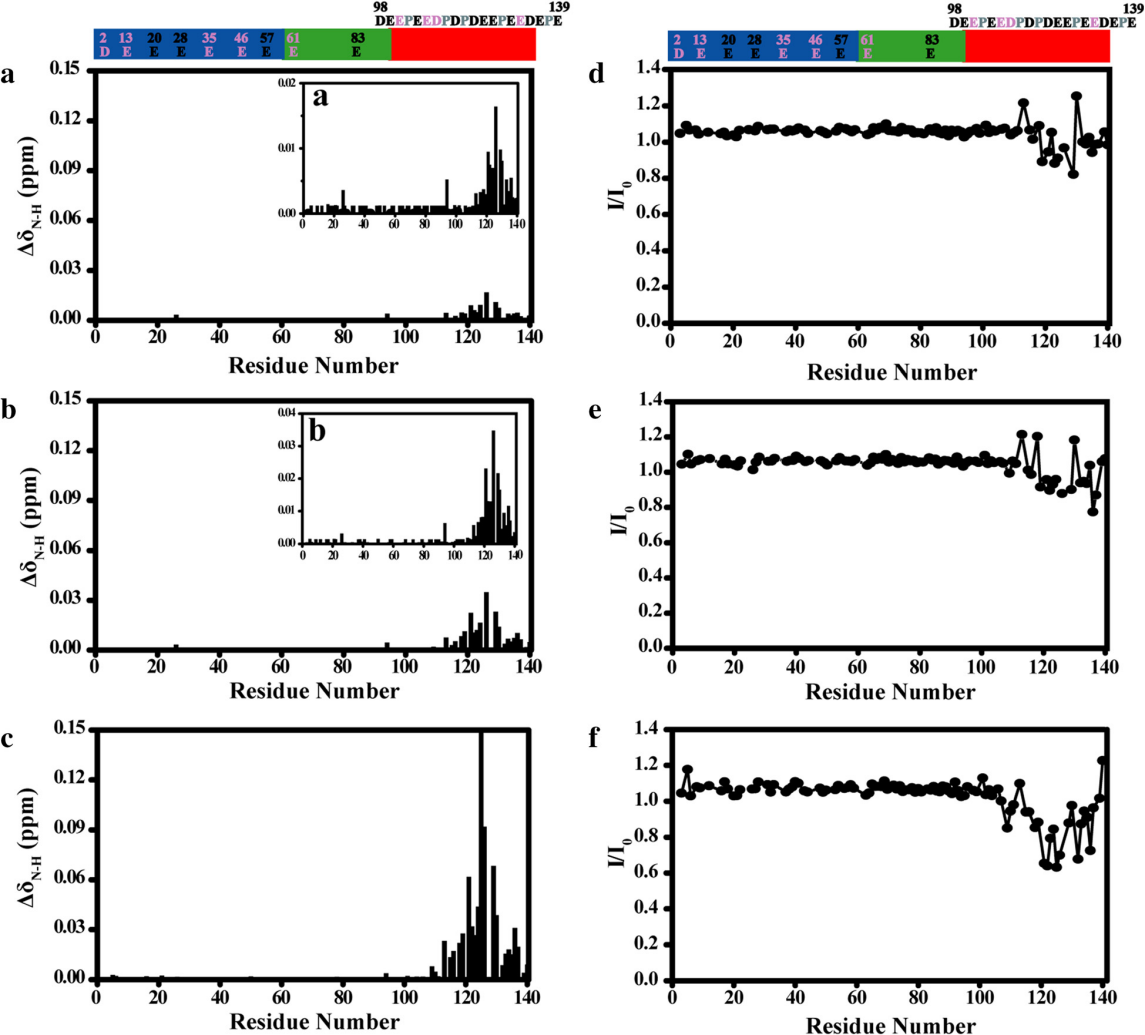
Chemical shifts and intensities changes of amide groups in αS at various concentration of Lu^3+^. The Δδ_N-H_ of α-synuclein backbone amide groups were plotted as a function of residue number at molar ratios of αS/Lu^3+^ (**a**) 4/1, (**b**) 2/1, (**c**) 1/1, respectively. Inserts (**a**), (**b**) in (**a**) and (**b**) with smaller vertical scale. I/I_0_ profiles of α-synuclein backbone amide groups were plotted as a function of residue number at molar ratios of αS/Lu^3+^ (**d**) 4/1, (**e**) 2/1, (**f**) 1/1, respectively. αS has three distinct regions that were shown in different colour: the N-terminus (residues 1–60) is shown in blue; the hydrophobic NAC part (residues 61–95) is shown in green; and the C-terminus (residues 96–140) is shown in red. Asp, Glu residues in the C-terminus include D98, E104, E105, E110, E114, D115, D119, D121, E123, E126, E130, E131, D135, E137, E139, respectively, and Asp, Glu residues in the other regions were also marked in the figure. Pro residues marked in gray had no cross peaks in the ^1^H-^15^N HSQC spectra. Residues highlighted in pink had no assignments in ^1^H-^15^N HSQC spectra

To map the binding sites of Lu^3+^ in αS, the chemical shift perturbations Δδ_N-H_ induced by Lu^3+^ were plotted as a function of αS sequence. As shown in Fig. 2, significant chemical shift perturbations were located from residue 110 to 140 in the C-terminal region and residue Y125 with the largest chemical shift perturbation, whereas small or no change was observed for the residues in the N-terminal and NAC regions. With the molar ratio of αS/Ln^3+^ decreased from 4/1 to 1/1, the chemical shift perturbation of residues at the C-terminal region became larger, suggesting that Lu^3+^ binding with αS in the C-terminal region centered at residue Y125.

The intensity ratios of cross peaks in the presence (I) and absence of Lu^3+^ (I_0_) were also shown in Fig. 2d-f. When the molar ratio of Lu^3+^/αS decreased from 4/1 to 1/1, the signal intensity of residues located in the C-terminal region decreased, whereas small or no change was observed for the residues in the N-terminal and NAC regions. We also noticed that many cross peaks of C-terminal residues disappear when αS/Lu^3+^ ratio decreased further to 1/10 (Additional file 1: Figure S1), suggesting that binding with Lu^3+^ slows down αS conformational exchange rate.

**Fig. 3:**
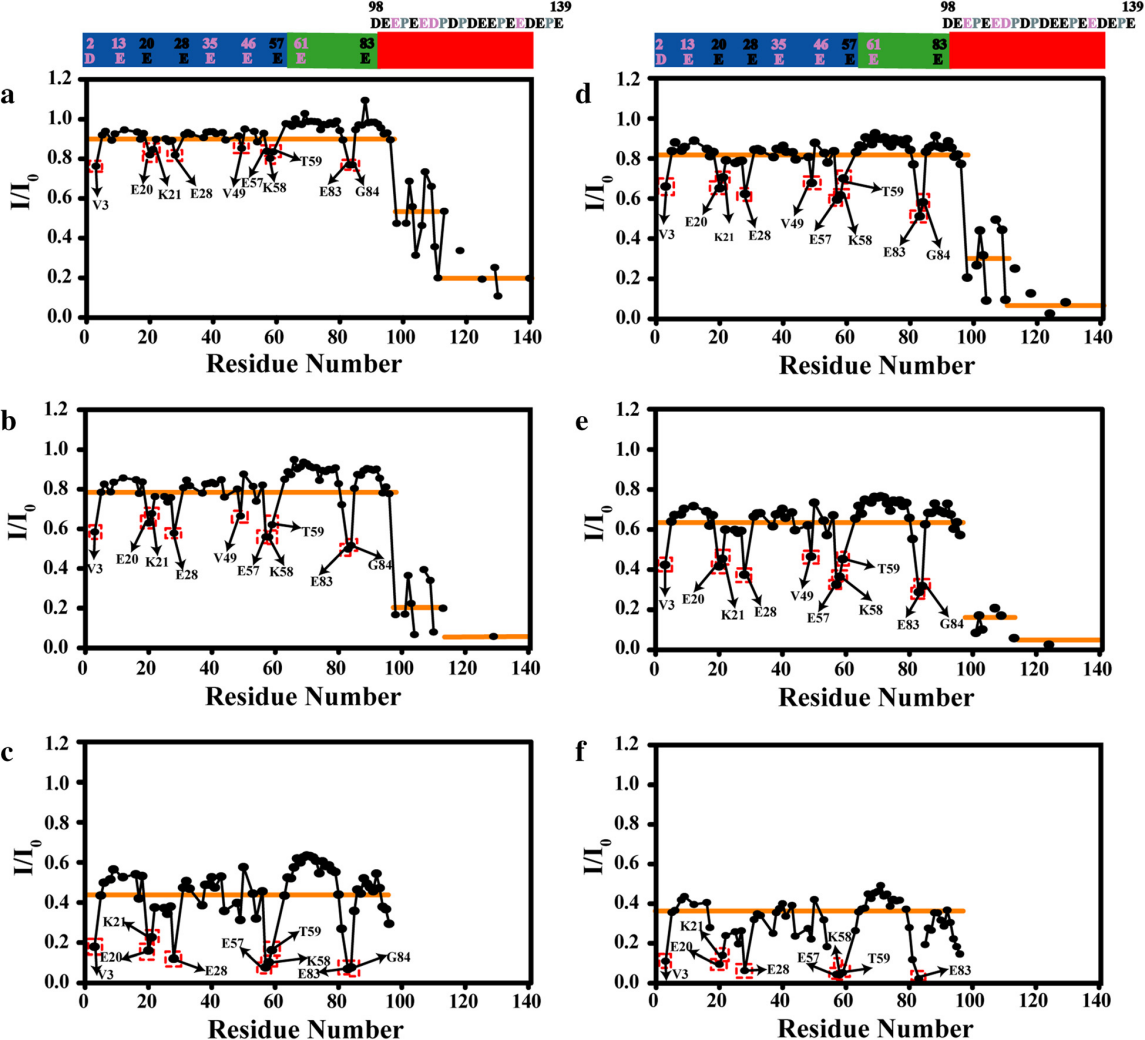
Intensities changes of amide groups in αS at various concentration of Tb^3+^ or Dy^3+^. The I/I_0_ profiles of α-synuclein backbone amide groups were plotted as a function of residue number at molar ratios of (**a**) αS/Tb^3+^(4/1), (**b**) αS/ Tb^3+^(2/1), (**c**) αS/Tb^3+^(1/1), (**d**) αS/Dy^3+^(4/1), (**e**) αS/Dy^3+^(2/1), (**f**) αS/Dy^3+^ (1/1), respectively. The residues that were severely broadened were labelled in red rectangle. According to the attenuation degree of cross peaks (I/I_0_), the whole residues of αS were divided into three parts: residues 1 to 97, residues 98 to 109, and residues 110 to 140, which were all labelled with orange lines. αS sequence shown above the figure is the same as that in Fig. 2

In contrast to diamagnetic Lu^3+^, we did not observe residues with significant chemical shift change in the presence of the paramagnetic ions (Tb^3+^, Dy^3+^). By analysing the ^1^H-^15^N HSQC spectra, we found that some cross peaks showed significant intensity decrease and some cross peaks were even too broad to detect as a result of paramagnetic effects of Tb^3+^ and Dy^3+^. We plot the intensity ratios (I/I_0_) of cross peaks in the presence (I) and absence of Tb^3+^ (or Dy^3+^) (I_0_) as a function of αS sequence. As shown in Fig. 3, the intensity of C-terminal residues decreased much more than that of residues in the N-terminal and NAC region. A comparative analysis with Tb^3+^ and Dy^3+^ revealed a hierarchy in the binding of lanthanide ions to αS. According to the attenuation profile of cross peaks (I/I_0_), the whole residues of αS were divided into three parts: residues 1 to 97, residues 98 to 109, and residues 110 to 140. It is clear that the lanthanide ions bound preferentially to the region comprising residues 110–140, the secondary binding regions may contain residues 98–109, and the weak interaction binding regions contained residues in 1–97 regions, which were labelled with orange lines in Fig. 3. With the molar ratio of αS/Ln^3+^ decreased from 4/1 to 1/1, the I/I_0_ values of all the residues other than the residues in the C-terminal region decreased, while the I/I_0_ values of residues 3, 20, 21, 28, 57, 58, 59, 83, 84 decreased more than other residues in the N-terminal and NAC regions. We found these residues are aspartic acid and glutamic acid residues or residues near to aspartic acid and glutamic acid (Fig. 3). Interestingly, they are all negatively charged residues that have a carboxyl groups in the side-chain, which may have transient weak interactions with positively charged lanthanide ions. αS C-terminus contains many aspartic acid and glutamic acid residues and it was reasonable that lanthanide ions bound preferentially to this region.

We also used ^1^D ^1^H spectra to obtain information on the roles of αS aromatic side chains in lanthanide binding. The ^1^H NMR spectra of αS in D_2_O (6.3-7.5 ppm) comprised the side chains of different aromatic residues: Phe (F4, F94), Tyr (Y39, Y125, Y133, Y136) (Additional file 1: Figure S2 and Figure S3), and the signals were assigned according to previous reports [27]. In the presence of low concentration lanthanide ions (Tb^3+^ and Dy^3+^), the Tyr signals intensity decrease significant, and with lanthanide ions (Tb^3+^ and Dy^3+^) concentration increasing, the Phe signals were further affected, suggesting lanthanide ions bound preferentially to C-terminus and might have transient weak interactions with N-terminal and NAC regions, consistent with ^1^H-^15^N HSQC spectra.

Many other metal ions and polyamines [42] also bind to the αS C-terminal region from residue 110 to 140. Other than the C-terminus, weaker binding sites involved residues 46–55 in the αS N-terminus was also reported for paramagnetic divalent cations such as Fe^2+^ and Co^2+^[27]. For Cu^2+^, the specific binding sites were identified as residues Met1, Asp2, and His50 in the N-terminus [27, 30]. It was worth mentioning that the binding sites of Ln^3+^ involved residues different from those binding with other divalent cations [27, 28, 30]. Residues containing a carboxyl groups in the N-terminal and NAC regions also have transient weak interaction with lanthanide ions. The binding character difference might be due to the inherent properties of different metal ions, such as charge, ionic radii and coordination abilities. The lanthanide metal ions, which possess ionic radii similar to Ca^2+^, are assumed to behave very similar to Ca^2+^ in protein binding. Through NMR titration experiment, we found that Ca^2+^ binding sites located in the αS C-terminus (Additional file 1: Figure S4 and Figure S5). The chemical shift perturbations of C-terminal residues are much smaller in the presence of Ca^2+^ than that of Lu^3+^, suggesting that αS-Ca^2+^ binding is relatively weak. The pattern of the chemical shift perturbation was also different from that induced by lanthanides ions. Our observations suggest that although lanthanides ions have similar ionic radii to Ca^2+^, they show different binding properties with αS. Diamagnetic metal ion Al^3+^ was also reported as an effective promoter to αS fibrillation, and we also studied Al^3+^ binding sites in αS (Additional file 1: Figure S6 and Figure S7). We found no obvious chemical shift perturbations were observed in the presence of Al^3+^, and only large intensity attenuation of cross peaks were located in the αS C-terminus, which suggested that binding with Al^3+^ slows down αS conformational exchange rate. Such conformational exchange was also observed in the presence of high concentration of Lu^3+^ (Additional file 1: Figure S1).

Since the affinities for the Ln^3+^ with αS is similar, the different degree of broadening for Dy^3+^ and Tb^3+^ must be from slightly different paramagnetic properties: the total angular momentum quantum number J for Dy^3+^ is 15/2, which is a little larger than that for Tb^3+^ (J = 6), meanwhile the unpaired electron correlation time τ_e_ of Dy^3+^ is 0.3 × 10^−12^s, which is a little larger than that of Tb^3+^ (τ_e_ = 0.2 × 10^−12^s) [43]. These different properties may explain why the intensity ratios of cross peaks in the presence of Dy^3+^ decrease more severely than that in the presence of Tb^3+^. The strong paramagnetic properties of lanthanide ions might have solvent PRE effect, which makes the cross peaks intensity ratios of most observed residues in the N-terminal and NAC region decreased to 0.4 or 0.6 (Fig. 3c, f).

As for diamagnetic ion (Lu^3+^), significant chemical shift perturbations were not observed in the N-terminal and NAC regions. It may be due to the fact that PRE is more sensitive to local structural perturbation than chemical shift, because paramagnetic relaxation enhancement is proportional to r^−6^, where r is the distance between paramagnetic ion and nucleus observed.

**Fig. 4:**
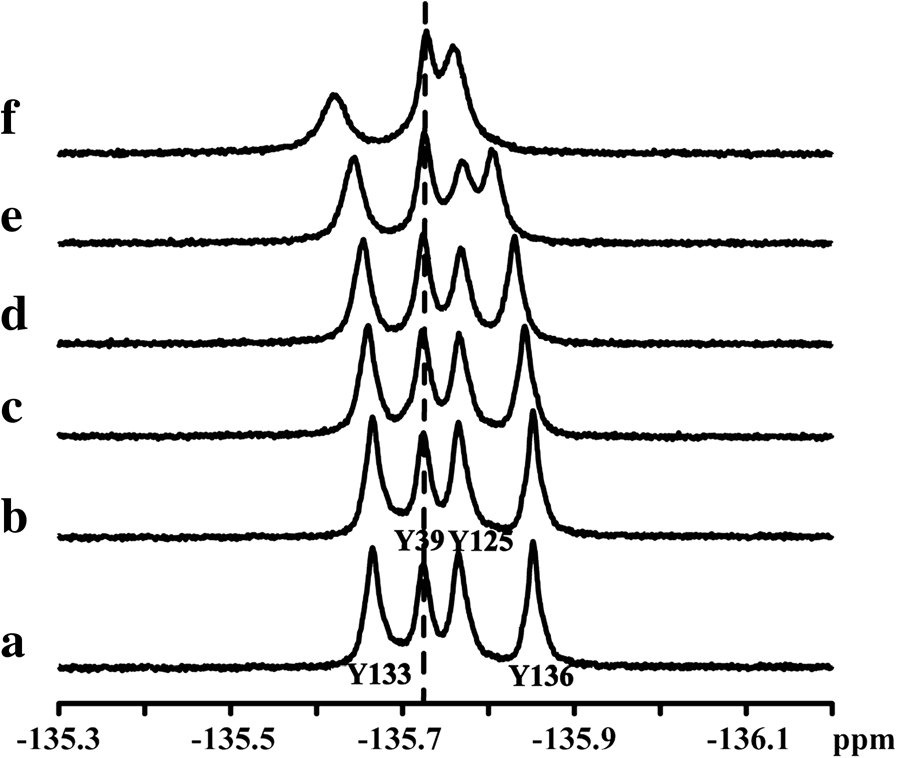
1D ^19^F spectra of α-synuclein at different molar ratios of αS/Lu^3+^ (**a**) 1/0, (**b**) 4/1, (**c**) 2/1, (**d**)1/1, (**e**) 1/2, (**f**) 1/4

To further confirm the binding sites, ^19^F NMR was also employed to study the αS - Lu^3+^ interaction. The ^19^F chemical shift is sensitive to the change of local chemical environment, hence it is a good reporter of binding sites. 3-^19^F tyrosine labelled αS were prepared and ^1^D ^19^F NMR spectra were recorded at different concentration ratios of Lu^3+^ (Fig. 4). The four ^19^F resonances (tyrosine 133, 39, 125, 136) were assigned according to previous research [44]. From Fig. 4, the ^19^F chemical shift of residue 39 in the N-terminus remained the same at different αS/Lu^3+^ ratios, however, chemical shift and shape of residues 125, 133, 136 in the C-terminal region changed during the titrations, indicating the binding is in the fast time scale and is weak as the titration is incomplete. The results suggested that the primary binding sites of Lu^3+^ located at C-terminal region, which was consistent with ^1^H-^15^N HSQC and 1D ^1^H experimental results. In a word, like many divalent metal ions such as Fe^2+^, Mn^2+^, Co^2+^, Ni^2+^ and Ca^2+^ [27, 28], lanthanide ions also bound non-specifically to the C-terminal domain of αS, and residues contain the carboxyl groups in the N-terminal and NAC regions that also have transient weak interaction with lanthanide ions, which was not reported in other studies of divalent metal ions interacting with αS.

### Lanthanide metal ions effects on αS conformation

**Fig. 5:**
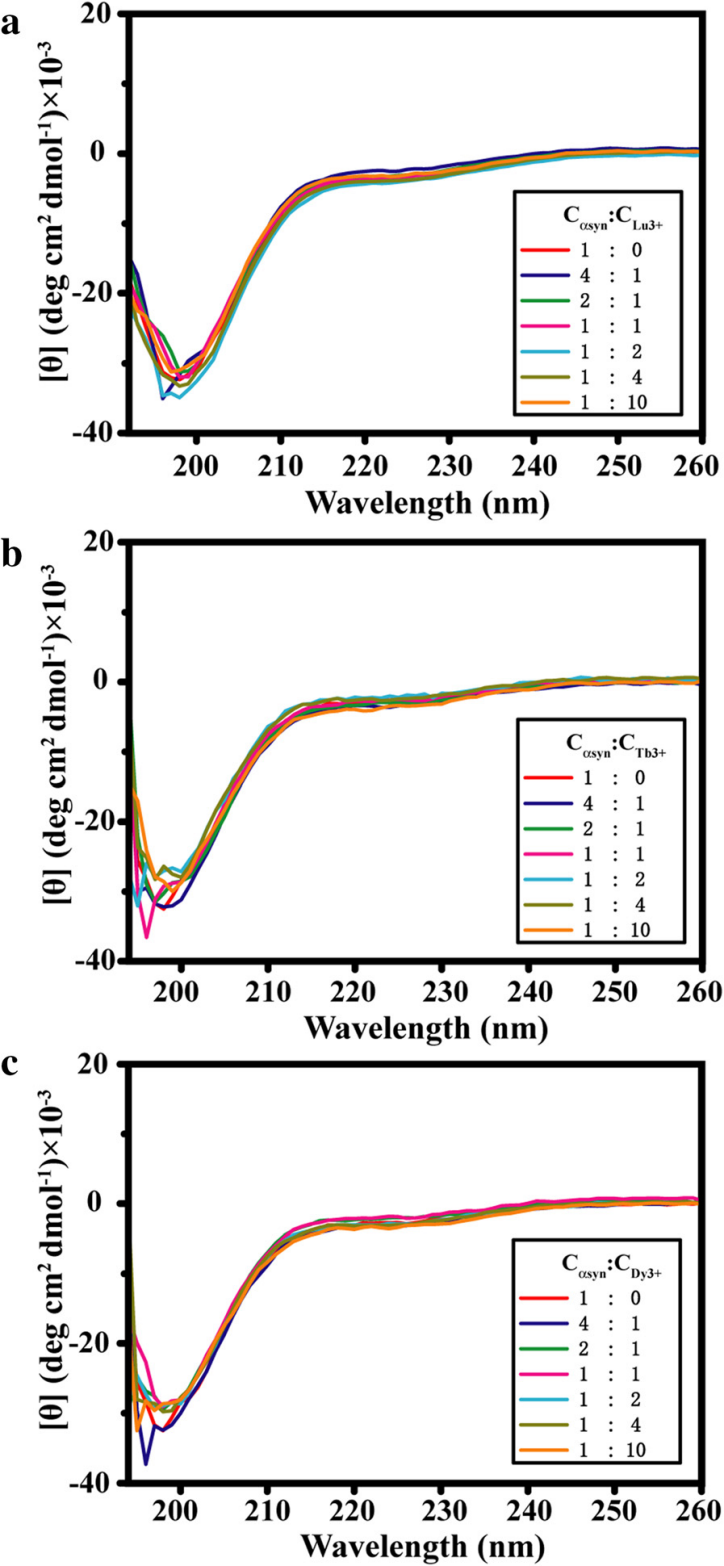
Far-UV CD spectra of α-synuclein at different molar ratios of lanthanide metal ions, (**a**) αS/Lu^3+^, (**b**) αS/Tb^3+^, (**c**) αS/Dy^3+^, respectively

The narrow dispersion (7.8-8.3 ppm) of amide ^1^H chemical shift suggested that αS remained disordered in the presence of lanthanide metal ions. Besides NMR spectroscopy, far-UV circular dichroism (CD) was also used to monitor the conformational change in presence of lanthanide metal ions. In the absence of Ln^3+^, the spectra of αS exhibited substantially disordered structure (no characteristic α-helix or β-sheet peaks in the 210–230 nm or 198 nm regions). In the presence of different ionic ratios, even in the higher ionic ratios, the spectra were always indicative of essentially unfolded protein, suggesting that lanthanide metal ions binding could not cause significant structural change of αS (Fig. 5).

The hydrodynamic property of a macromolecule has been extensively used to study conformational changes accompanying molecular unfolding, association and aggregation. We employed ^1^H pulsed-field gradient NMR to investigate the hydrodynamic properties of the αS-Lu^3+^ complexes at different concentration ratios of αS/Lu^3+^. We measured the hydrodynamic radiuses of αS in the absence and presence of Lu^3+^. The radius of αS in the free state was approximately 31 Å, consistent with previous reports [27], and no large changes were observed at different αS/Lu^3+^ concentration ratios (Table 1). It suggested that binding with Lu^3+^ did not affect αS size, or cause any inter-molecular association.

**Table 1:** The measured translational diffusion coefficients of dioxane and α-synuclein and the calculated hydrodynamic radius of α-synuclein at different concentration ratios of αS/Lu^3+^

C_αS_ : C_Lu3+_	Translational diffusion coefficients		Hydrodynamic radius R_H_ (Å)
	αS (methyl regoins from 0.87–0.56 ppm) *D*_t_ × 10^−11^(m^2^/s)	Dioxane (3.65–3.53 ppm) *D*_t_ × 10^−10^(m^2^/s)	
1 : 0	5.802 ± 0.001	8.482 ± 0.004	30.99 ± 0.05
4 : 1	5.949 ± 0.004	8.610 ± 0.004	30.68 ± 0.03
2 : 1	5.823 ± 0.006	8.488 ± 0.004	30.90 ± 0.04
1 : 1	5.845 ± 0.004	8.575 ± 0.004	31.10 ± 0.03
1 : 2	5.939 ± 0.003	8.626 ± 0.004	30.79 ± 0.02
1 : 4	5.915 ± 0.003	8.694 ± 0.005	31.16 ± 0.02

### Lanthanide metal ions effects on αS fibrillation

**Fig. 6:**
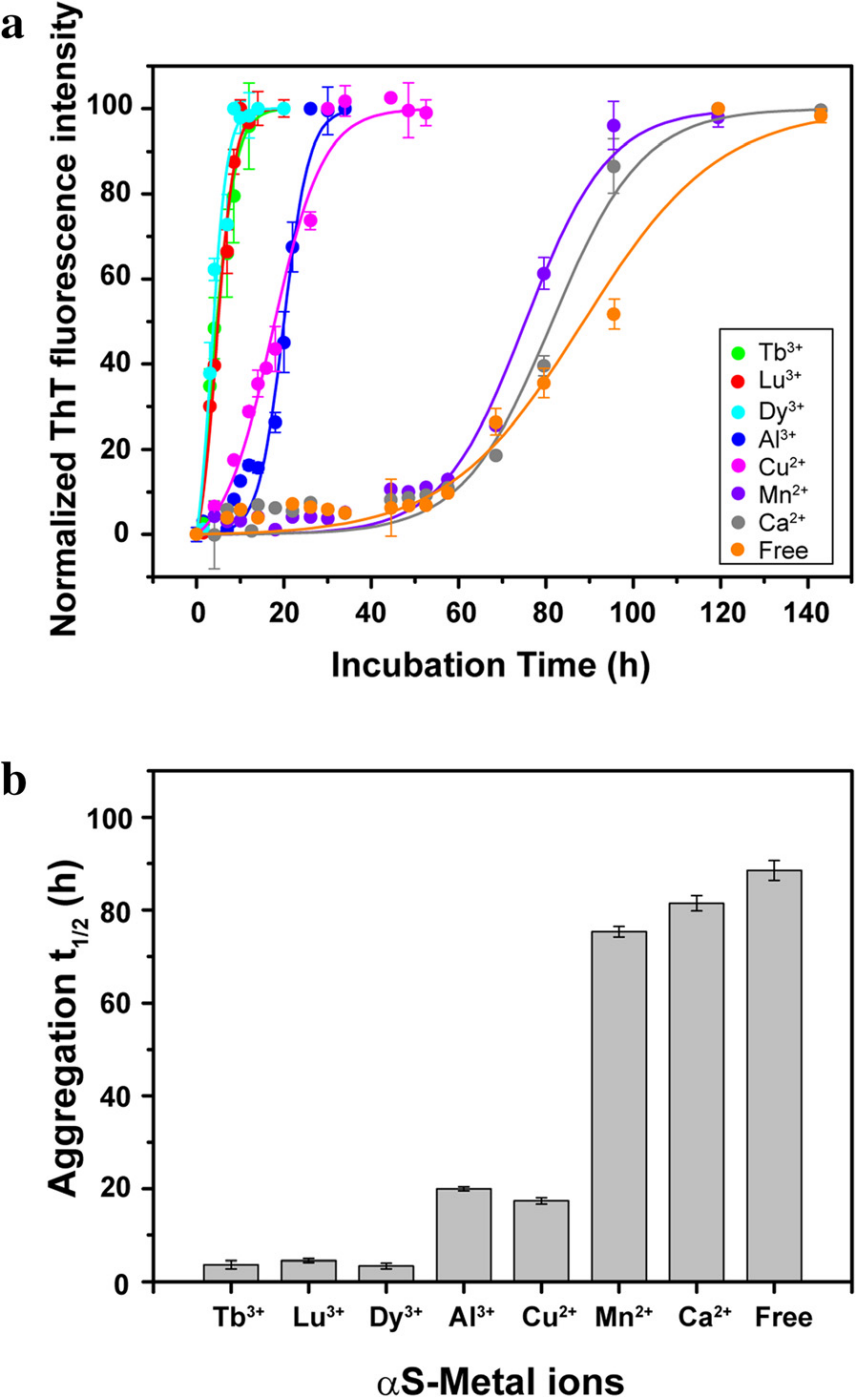
**a** Comparison of αS fibrillation rate in the presence of different metal ions. 100 μM αS in the presence of 0 μM metal ions (*orange*), 400 μM Ca^2+^ (*gray*), Mn^2+^ (*violet*), Cu^2+^ (*magenta*), Al^3+^ (*blue*), Dy^3+^ (*cyan*), Lu^3+^ (*red*), Tb^3+^ (*green*), respectively. Fluorescence intensity was normalized to 100 units. The error bars in figure were standard deviations of three measurements. Points with no visible error bars represent measurements with tiny variance. Solid lines were the fits according to equation 1. **b** Lag time t_1/2_ values (hours) obtained for αS in the absence and presence of different metal ions: control (89 ± 2), Ca^2+^ (82 ± 2), Mn^2+^ (75 ± 1), Cu^2+^ (17 ± 1), Al^3+^ (20 ± 1), Dy^3+^ (4 ± 1), Lu^3+^ (5 ± 1), Tb^3+^ (4 ± 1). Error bars denote the fitting errors of experiments

αS formed insoluble amyloid fibrils under some certain experimental conditions, and Thioflavin T (ThT) fluorescence was generally used to monitor its fibrillation [45–48]. We studied αS fibrillation rates at different concentration ratios of αS to lanthanide metal ions (Additional file 1: Figure S8). The results indicated that αS fibrillation was accelerated in the presence of Ln^3+^, and the accelerating effect of Ln^3+^ depend on its concentration, higher concentration resulted in faster fibrillation. Different lanthanide metal ions seemed to have similar effects on promoting αS fibrillation. Compared to cations such as Al^3+^, Cu^2+^ and Mn^2+^, lanthanide metal ions (Lu^3+^, Dy^3+^, Tb^3+^) induced αS fibrillation much faster (Fig. 6). Ca^2+^, which possess ionic radii similar to lanthanide ions, are assumed to behave very similar to lanthanide ions in αS fibrillation, but to our surprise, Ca^2+^ did not significantly accelerate αS fibrillation. This result suggests that αS fibrillation kinetics can be modulated by interactions.

Many metal ions can accelerate αS fibrillation. The reason is very complex, and many factors involved in it. The different effects on αS fibrillation rate might be due to the inherent properties of different metal ions, such as binding sites, coordination modes. Positively charged metal ions neutralizing negatively charged αS, and resulting in stronger intermolecular association is also regarded as a reason for αS faster aggregation. Besides binding with αS C-terminus, lanthanide metal ions show very novel coordination modes, and residues contain the carboxyl groups in αS N-terminal and NAC regions that also have transient weak interaction with lanthanide ions, which is not reported in other studies of divalent metal ions interacting with αS. This specific interaction can disturb αS local conformation, and may be another reason for αS faster fibrillation. We also notice that many cross peaks of C-terminal residues disappear when αS/Lu^3+^ ratio decreased further to 1/10, suggesting that the presence of Lu^3+^ reduces the αS conformation exchange rate. Such conformational exchange reduction is also observed in the presence of Al^3+^. Slow αS conformational exchange rates make the expose hydrophobic residues contact long enough to form associated oligomers, which is crucial for protein fibrillation. When the conformational exchange rate is fast or almost the same as the bimolecular encounter rate, there is not enough time for molecules association occurring.

## Conclusions

In summary, we identified the lanthanide metal ions binding sites in αS by employing the chemical shift perturbations and paramagnetic effects of these metal ions. In particular, we found a hierarchal effect for lanthanide ions binding to αS, driven by the interaction with specific residues, namely, aspartic acids, glutamic acids residues. Compared to divalent cations, lanthanide metal ions significantly accelerated αS fibrillation, possibly due to their different inherent properties such as charge, binding sites and coordination modes. The study here also suggest that binding induced change of conformational exchange dynamics provide a possible molecular mechanism to understanding αS fibrillation.

## Methods

### Protein expression and purification

Plasmids contained the coding sequence of α-synuclein were transformed into *Escherichia coli* strain BL21 (DE3) competent cells. Expression and purification of ^15^N-labeled wild-type or ^19^F-labeled (Y39F, Y125F, Y133F, Y136F) α-synuclein were performed as previously described [44, 49, 50].

### Chemical Reagents

Thioflavin T (ThT), TbCl_3_•6H_2_O, DyCl_3_•6H_2_O and LuCl_3_•6H_2_O from Sigma or Alfa Aesar were used without further purification. All other chemicals were of analytical grade from Sinopharm Chemical Reagent Co. Ltd.

### Circular dichroism (CD) measurements

Samples containing 20 μM αS with different concentration of lanthanide metal ions in 10 mM MES and 100 mM NaCl at pH 6.0 were used for CD measurements. CD spectra were recorded on a ChirascanTM CD spectrometer in 10 mm cells from 260-190 nm with a step size of 1 nm and a bandwidth of 1 nm.

### Fluorescence measurements

Samples comprised of 100 μM αS with 400 μM different metal ions in 10 mM MES, 100 mM NaCl, 500 μM phenylmethanesulfonyl (pH 6.0) were used for fibrillation experiments. αS fibril formation was induced at 37 °C by agitation with 220 rpm in the shaker. During fibrillation, small aliquots (10 μl) were removed and mixed with 1 mL 25 μM ThT. The fluorescence intensities were measured to monitor αS fibrillation. Fluorescence emission spectra were recorded at 482 nm on a HORIBA Fluoromax-4 spectrometer.

The aggregation data was analysed using equation (1) [42], where α[t] denotes the values measured by ThT fluorescence experiments, *k*_app_ is the apparent fibrillation rate constant, and t_1/2_ is the lag time to the transition midpoint in the process from monomer to aggregate.

1 $$ \alpha \left[t\right]=\left(1-{e}^{-{k}_{app}t}\right)/\left(1+{e}^{-{k}_{app}\left(t-{t}_{1/2}\right)}\right) $$

### NMR Spectroscopy and Data Analysis

NMR Buffer contained 10 mM MES, 100 mM NaCl (pH 6.0). Protein was concentrated to 0.25 mM in 90 % NMR Buffer and 10 % D_2_O. ^1^H-^15^N HSQC spectra and diffusion experiments were acquired on a Bruker 600 MHz spectrometer equipped with a triple-resonance cryoprobe at 288 K. The HSQC spectra were acquired with sweep widths of 7212 Hz (^1^H) and 1581Hz (^15^N), 2048 × 256 complex points, 4 scans per t_1_ point, and a 1.5 s recycle delay. The spectral widths for the diffusion experiments were 9615Hz, with 32 K complex points, 56 scans, and a 1 s recycle delay. ^1^D ^19^F NMR spectra were acquired on a Bruker 600 MHz spectrometer equipped with a ^19^F/^1^H/^13^C/^15^N cryoprobe. Spectra were recorded with a 11.3 kHz sweep width, 350 scans, 16 K complex points and a recycle delay of 2 s. Proton decoupling was applied to all ^1^D ^19^F NMR spectra. Chemical shifts are referenced to trifluorotoluene at −63.72 ppm. For analysis of the chemical shift perturbations (Δδ_N-H_) of ^1^H and ^15^N backbone resonances in the HSQC spectra, a weighted average chemical shift change were calculated according to the equation (2) [51], where Δδ_H_ and Δδ_N_ denoted the chemical shift difference in the presence and in the absence of Ln^3+^ in the ^1^H dimension and ^15^N dimension, respectively.

2 $$ \boldsymbol{\Delta} {\boldsymbol{\updelta}}_{\mathbf{N}-\mathbf{H}}=\sqrt{\frac{{\left(\boldsymbol{\Delta} {\boldsymbol{\updelta}}_{\mathbf{H}}\right)}^2+{\left(0.2\left(\boldsymbol{\Delta} {\boldsymbol{\updelta}}_{\mathbf{N}}\right)\right)}^2}{2}} $$

For the ^1^H pulsed-field gradient NMR experiments, we used 1,4-dioxane as an internal radius standard (2.12 Å) and viscosity probe. Diffusion spectra were acquired using the stimulated echo experiment with 3-9-19 pulse module for water suppression (stebpgp1s19). 20 linearly spaced values of gradient strength were used in the range from 0.963 to 45.74 G/cm. The optimized diffusion (Δ) and gradient pulse times (δ) were 0.45 s and 3 ms, respectively. Decays rates had been extracted from the spectra for both the protein (D_prot_) and the reference molecule (D_ref_), and the measured translational diffusion coefficients were showed in Table 1. The regions 0.87–0.56 ppm were used to calculate αS hydrodynamic radius (R_h_) according to the equation (3) [52], where R_h_^prot^ was the protein hydrodynamic radius and R_h_^ref^ was the radius of the reference molecule. Spectra were processed and analysed by Topspin [53], NMRPipe [54], and Sparky [55].

3 $$ {\mathbf{R}}_{\mathbf{h}}^{\mathbf{prot}} = \frac{{\mathbf{D}}_{\mathbf{ref}}}{{\mathbf{D}}_{\mathbf{prot}}}*{\mathbf{R}}_{\mathbf{h}}^{\mathbf{ref}} $$

## Availability of supporting data

All the supporting data are included as Additional file 1.

## Additional file

Additional file 1: Figure S1. Chemical shifts and intensities changes of amide groups in αS at various concentration of Lu^3+^. **Figure S2.** Tb^3+^ effects on ^1^D ^1^H spectra of αS aromatic side chains. **Figure S3.** Dy^3+^ effects on ^1^D ^1^H spectra of αS aromatic side chains. **Figure S4.** Ca^2+^ effects on αS ^1^H-^15^N-HSQC spectra. **Figure S5.** Chemical shifts and intensities changes of amide groups in αS at various concentration of Ca^2+^. **Figure S6.** Al^3+^ effects on αS ^1^H-^15^N-HSQC spectra. **Figure S7.** Intensities changes of amide groups in αS at various concentration of Al^3+^. **Figure S8.** Fibrillation of α-synuclein monitored by ThT fluorescence in the presence of different lanthanide metal ions. (DOC 1028 kb)

## Abbreviations

αS: α-synuclein
PD: Parkinson’s disease
NMR: Nuclear magnetic resonance
HSQC: Heteronuclear single quantum correlation
CD: Circular Dichroism
Ln^3+^: Lanthanide metal ions
ThT: Thioflavin T
MES: 2-(*N*-morpholino) ethanesulfonic acid
R_h_: Hydrodynamic radius
