# Supplementary material for: α-synuclein-lanthanide metal ions interaction: binding sites, conformation and fibrillation
Source: BMC Biophys. 2016 Feb 3;9:1. doi: 10.1186/s13628-016-0026-1 (PMC4739322; doi:10.1186/s13628-016-0026-1)
Supplement: Additional file 1: Figure S1. — Chemical shifts and intensities changes of amide groups in αS at various concentration of Lu3+. Figure S2. Tb3+ effects on 1D 1H spectra of αS aromatic side chains. Figure S3. Dy3+ effects on 1D 1H spectra of αS aromatic side chains. Figure S4. Ca2+ effects on αS 1H-15N-HSQC spectra. Figure S5. Chemical shifts and intensities changes of amide groups in αS at various concentration of Ca2+. Figure S6. Al3+ effects on αS 1H-15N-HSQC spectra. Figure S7. Intensities changes of amide groups in αS at various concentration of Al3+. Figure S8. Fibrillation of α-synuclein monitored by ThT fluorescence in the presence of different lanthanide metal ions. (DOC 1028 kb) [file 13628_2016_26_MOESM1_ESM.doc]

**Additional file 1 for**

α-synuclein-lanthanide metal ions interaction: binding sites, conformation and fibrillation

Jia Bai^1,2^, Zeting Zhang^1^, Maili Liu^1^, Conggang Li^1*^

1 Key Laboratory of Magnetic Resonance in Biological Systems, State Key Laboratory of Magnetic Resonance and Atomic and Molecular Physics, National Center of Magnetic Resonance in Wuhan, Wuhan Institute of Physics and Mathematics, Chinese Academy of Sciences, Wuhan 430071, P.R. China.

2 University of Chinese Academy of Sciences, Beijing 100049, China.

Jia Bai E-mail: baijiasx@126.com

Zeting Zhang E-mail: zhangzeting@wipm.ac.cn

Maili Liu E-mail: ml.liu@wipm.ac.cn

Conggang Li *Corresponding author E-mail: conggangli@wipm.ac.cn

**Figure S1:**


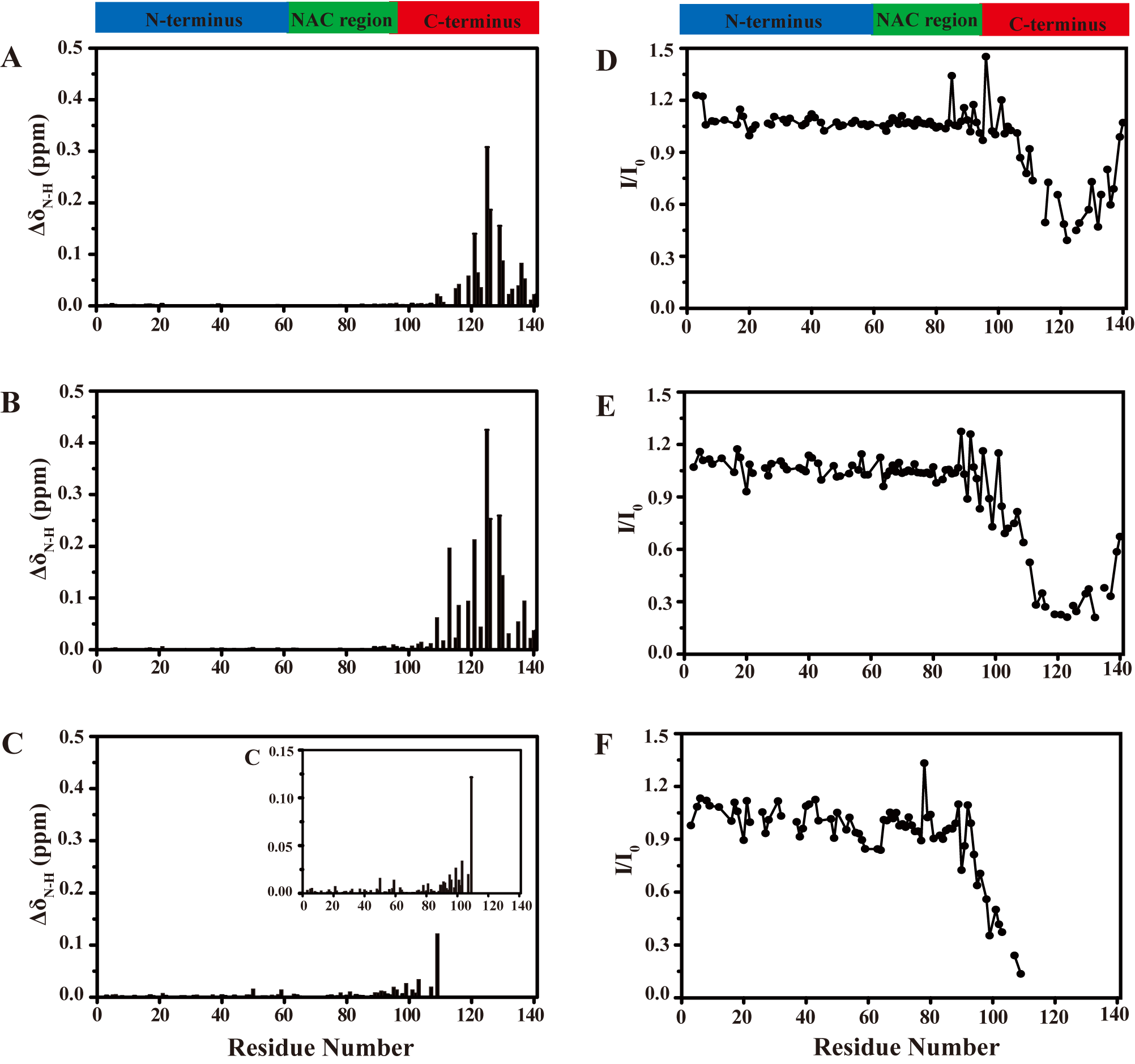


**Figure S1::** **Chemical shifts and intensities changes of amide groups in αS at various concentration of Lu^3+^**. The chemcial shift pertubation Δδ_N-H_ of αS backbone amide groups were plotted as a function of residue number at molar ratios of αS/Lu^3+^(A)1/2, (B)1/4, (C)1/10, respectively. Insert in (C) represented the same diagrams with small vertical scale. And the I/I0 profiles of α-synuclein backbone amide groups were plotted as a function of residue number at molar ratios of αS/Lu^3+^(D)1/2, (E)1/4, (F)1/10, respectively.αS has three distinct regions: the N-terminus (residues 1-60) was showed in blue; the hydrophobic NAC part (residues 61-95) was showed in green; and the C-terminus (residues 96-140) was showed in red.

**Figure S2:**


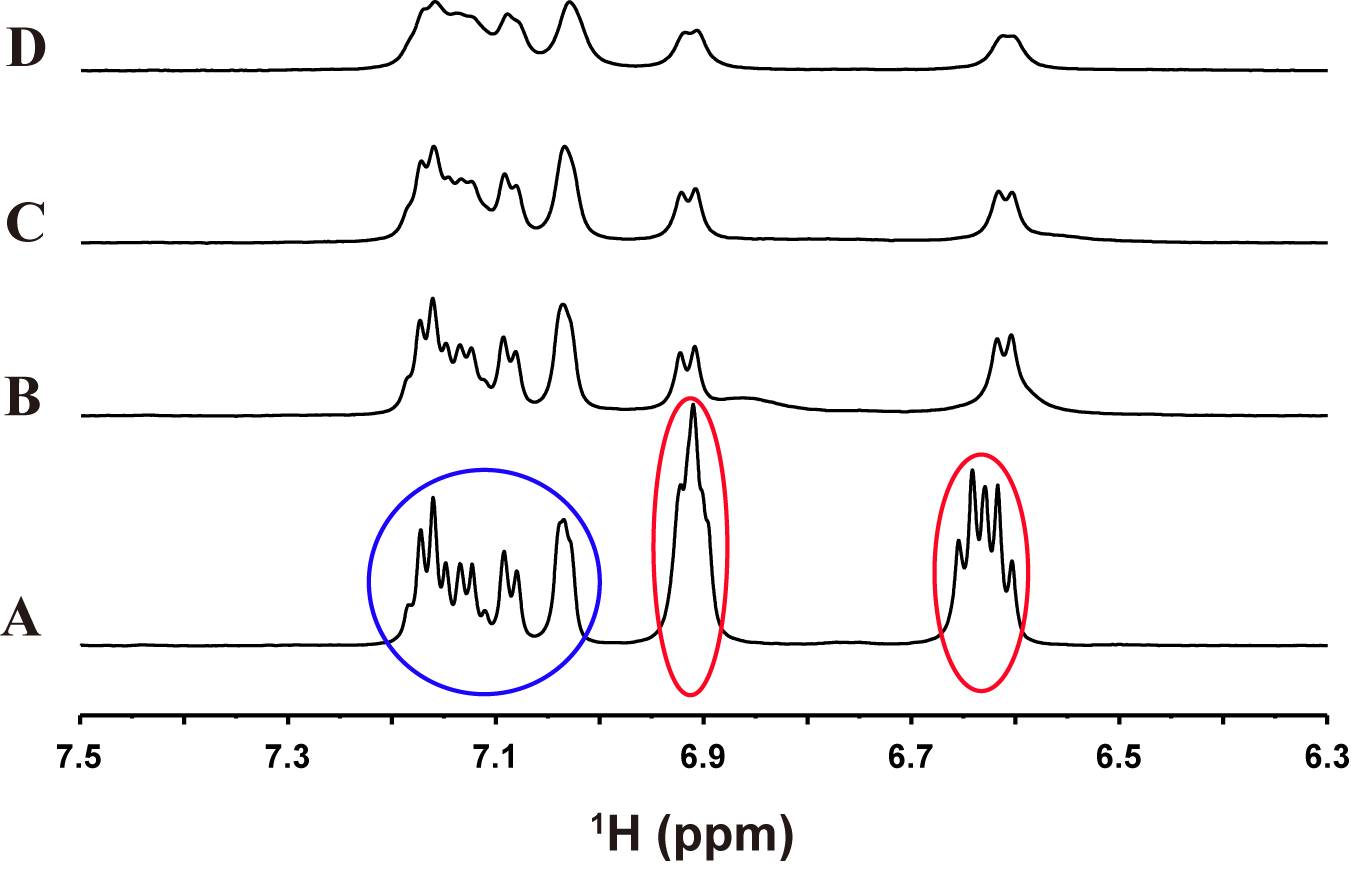


**Figure S2::** **Tb^3+^ effects on ^1^D ^1^H spectra of αS aromatic side chains.** All spectra were obtained at 15℃in D_2_O. Samples contained 100 μM αS in the (A) absence or presence of (B) 25 μM, (C) 50 μM, (D) 100 μM Tb^3+^, respectively. The signal of Phe (F4, F94) (blue) and Tyr (Y39, Y1125, Y133, Y136) (red) were marked in the figure.

**Figure S3:**


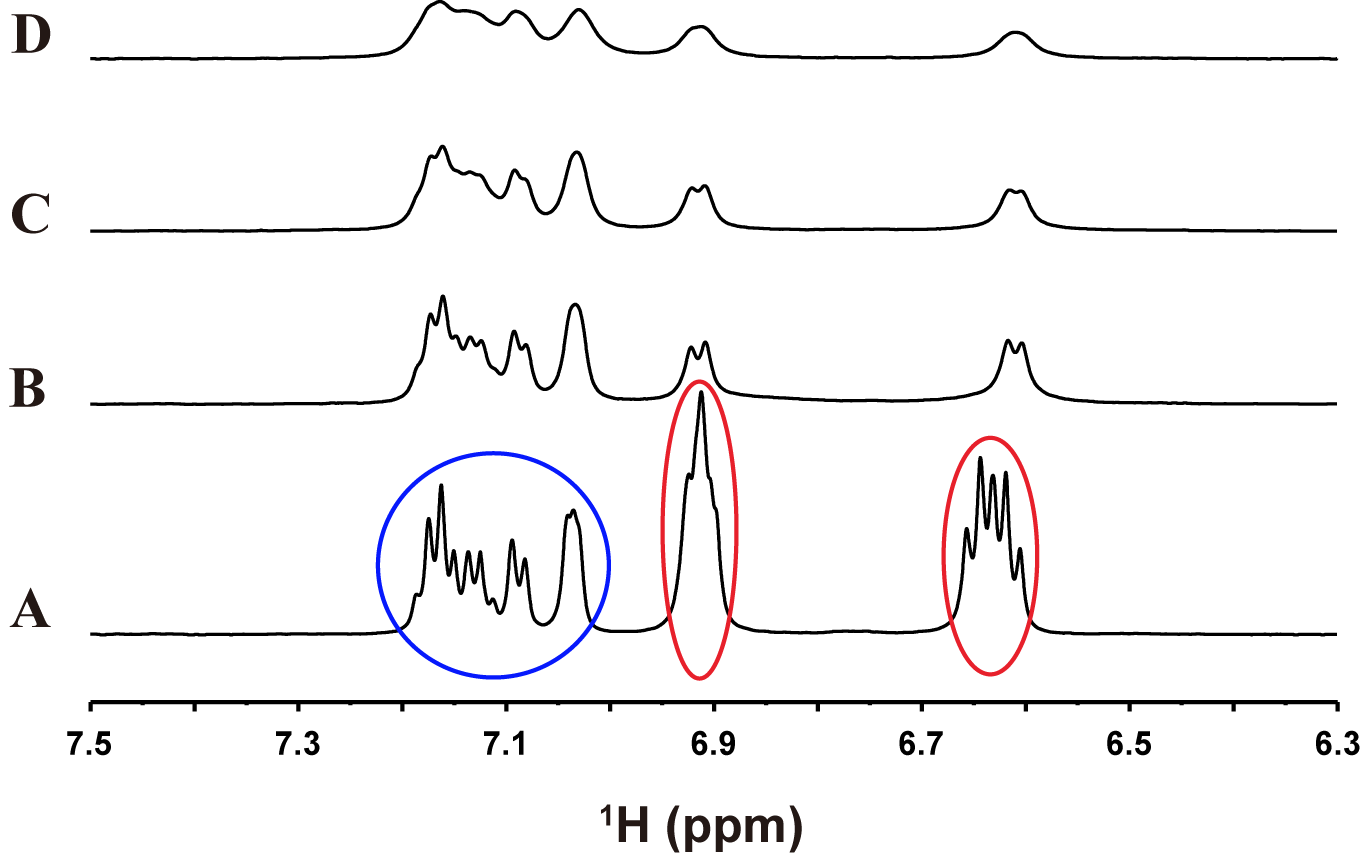


**Figure S3::** **Dy^3+^ effects on ^1^D ^1^H spectra of αS aromatic side chains.** All spectra were obtained at 15℃in D_2_O. Samples contained 100 μM αS in the (A) absence or presence of (B) 25 μM, (C) 50 μM, (D) 100 μM Dy^3+^, respectively. The signal of Phe (F4, F94) (blue) and Tyr (Y39, Y1125, Y133, Y136) (red) were marked in the figure.

**Figure S4:**


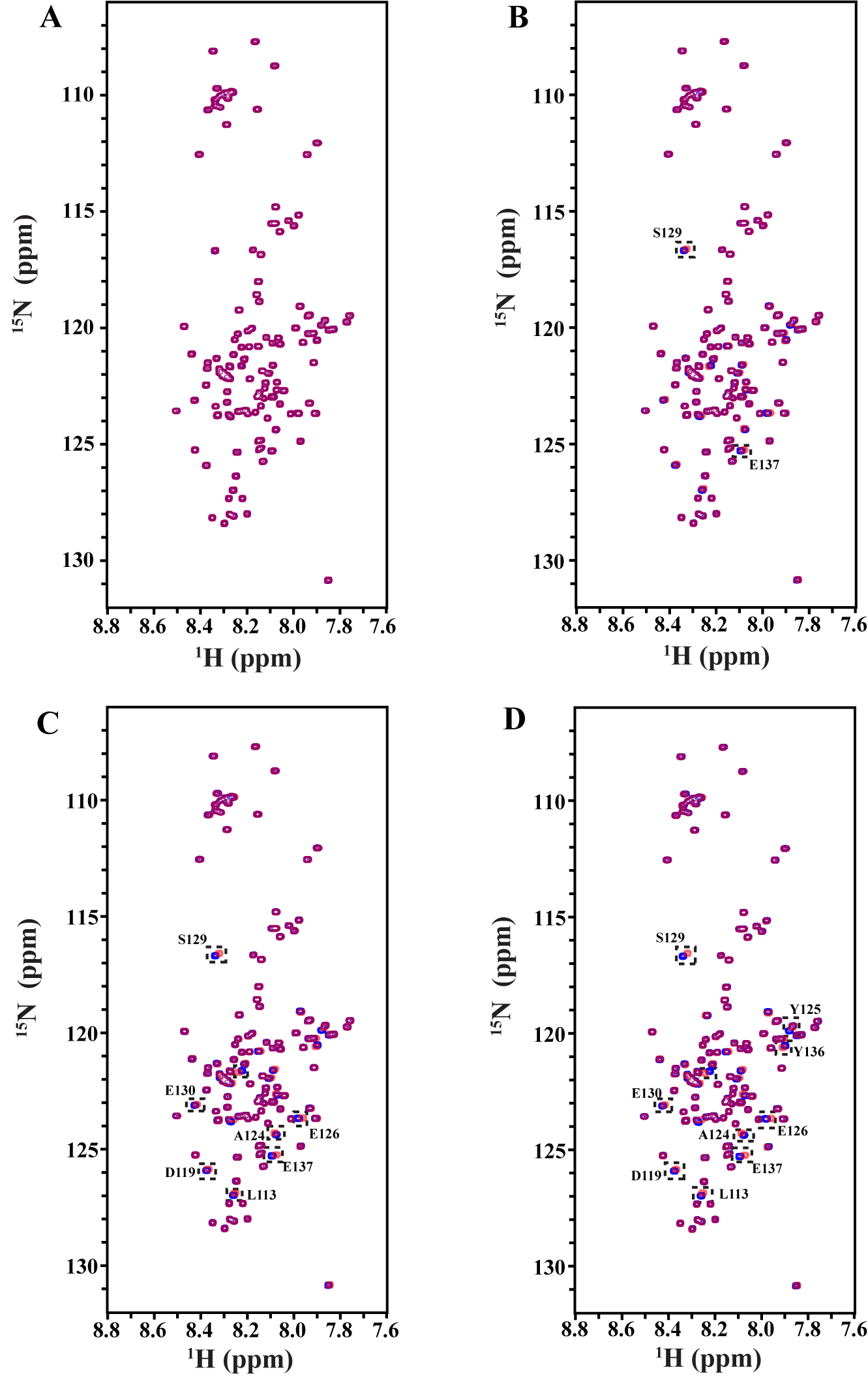


**Figure S4:**: **Ca^2+^ effects on αS ^1^H-^15^N-HSQC spectra.** ^1^H-^15^N HSQC of α-synuclein in the absence (blue) and presence of (red) molar ratios of αS/Ca^2+^ (A)1/1, (B)1/10, (C)1/20, (D)1/30, respectively. All spectra were obtained at 15℃. Residues with chemical shift change are labelled in the figure.

**Figure S5:**


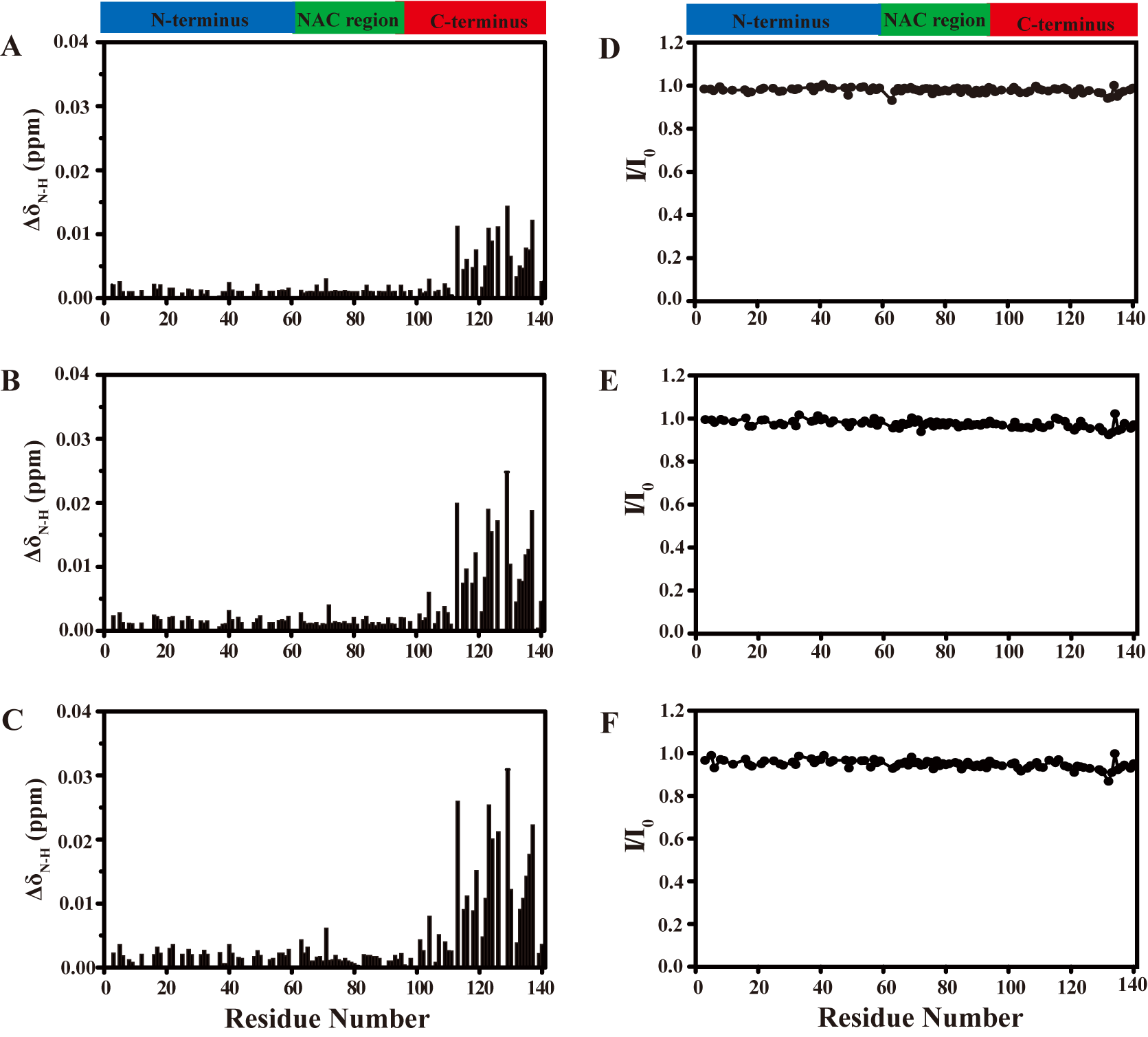


**Figure S5::** **Chemical shifts and intensities changes of amide groups in αS at various concentration of Ca^2+^.** The Δδ_N-H_ of α-synuclein backbone amide groups were plotted as a function of residue number at molar ratios of αS/Ca^2+^ (A)1/10, (B)1/20, (C)1/30, respectively. And the I/I_0_ profiles of α-synuclein backbone amide groups were plotted as a function of residue number at molar ratios of αS/Ca^2+^ (D) 1/10, (E) 1/20, (F) 1/30, respectively. αS has three distinct regions: the N-terminus (residues 1-60) was showed in blue region; the hydrophobic NAC part (residues 61-95) was showed in green region; and the C-terminus (residues 96-140) was showed in red region.

**Figure S6:**

**
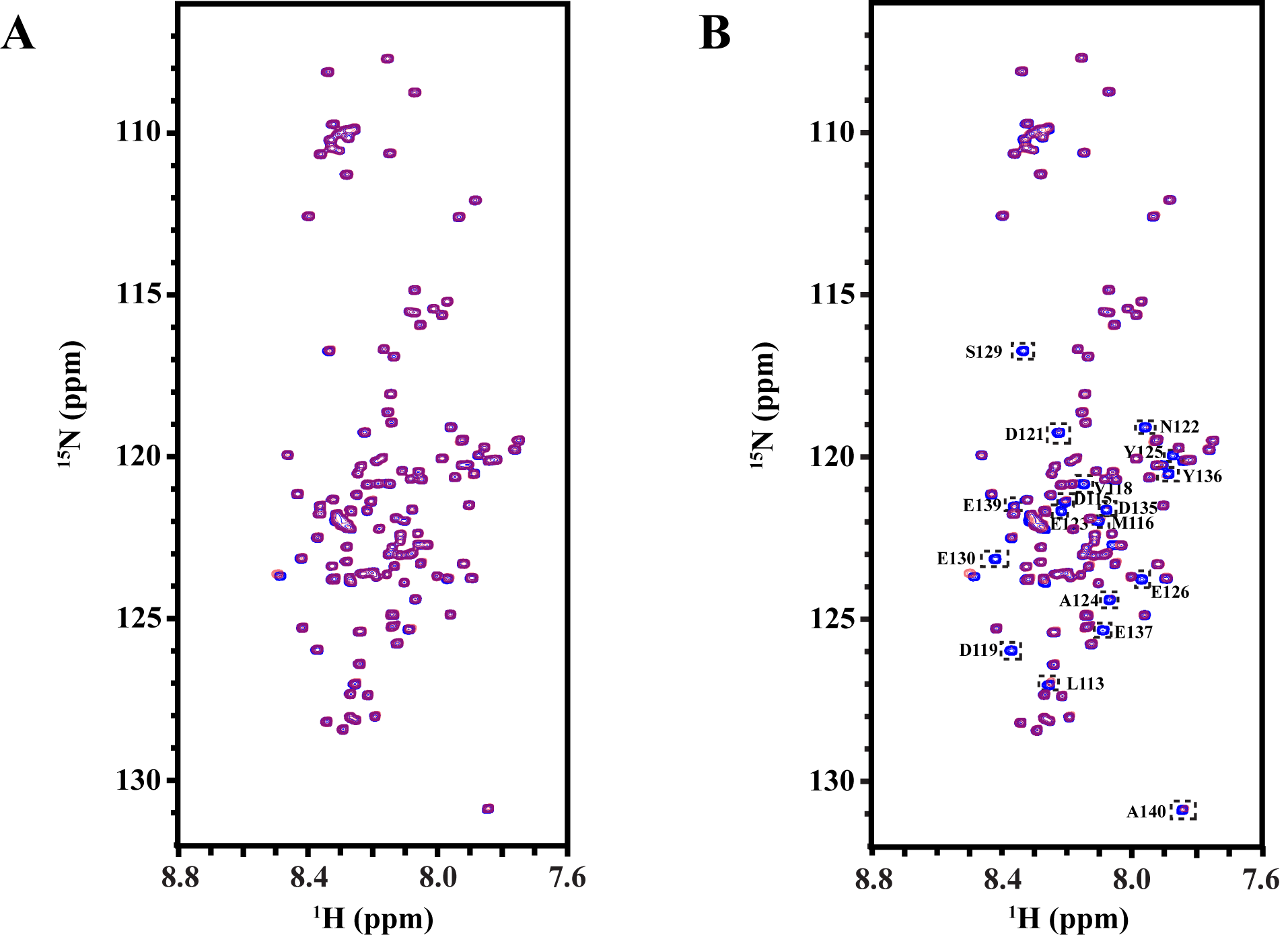
**

**Figure S6::** **Al^3+^ effects on αS ^1^H-^15^N-HSQC spectra.** ^1^H-^15^N HSQC of α-synuclein in the absence (blue) and presence of (red) molar ratios of αS/Al^3+^ (A)1/10, (B)1/20, respectively. All spectra were obtained at 15℃. Residues with chemical shift change are labelled in the figure.

**Figure S7::**

**
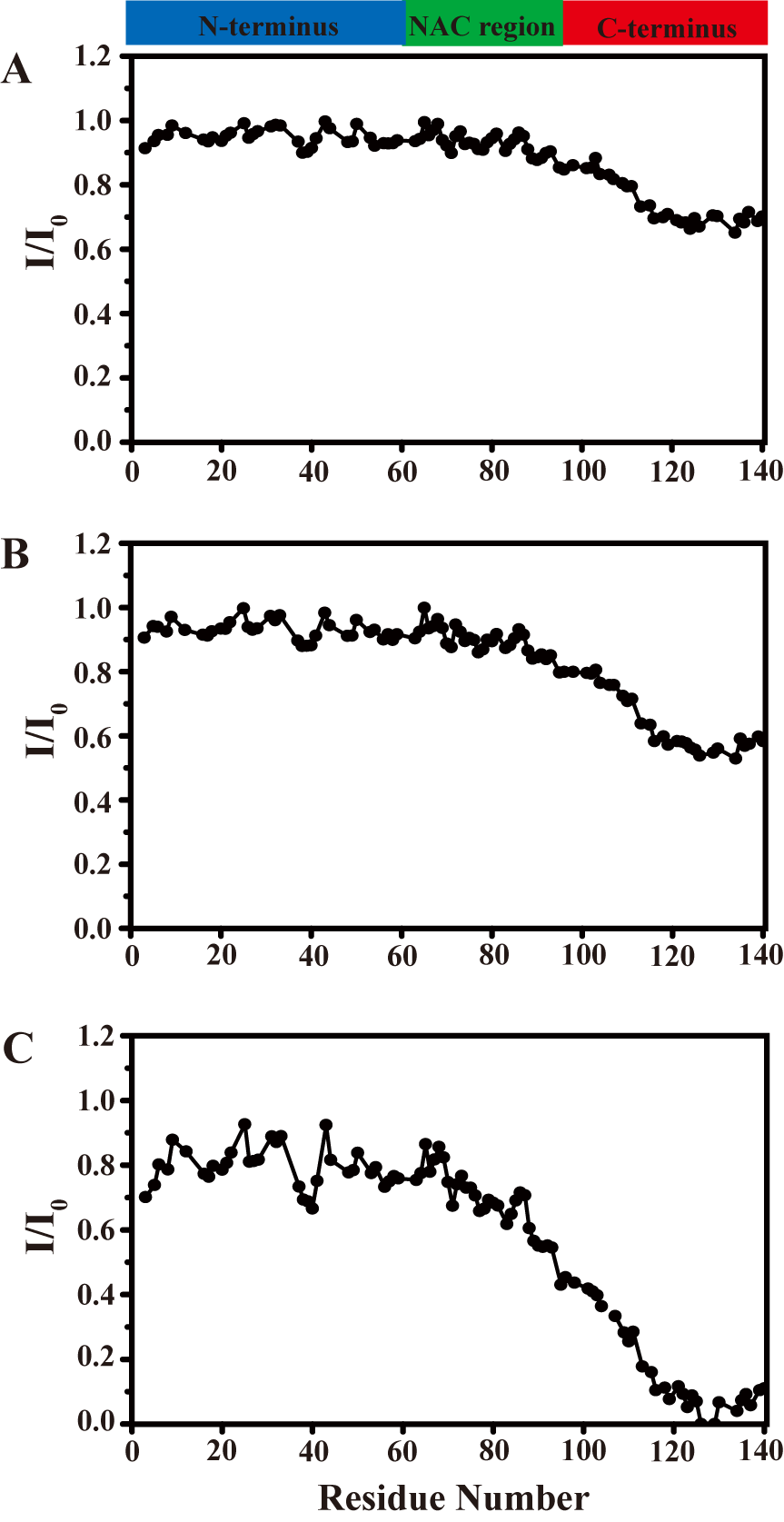
**

**Figure S7::** **Intensities changes of amide groups in αS at various concentration of Al^3+^.** The I/I_0_ profiles of α-synuclein backbone amide groups were plotted as a function of residue number at molar ratios of αS/Al^3+^ (A) 1/4, (B) 1/10, (C) 1/20, respectively. αS has three distinct regions: the N-terminus (residues 1-60) was showed in blue region; the hydrophobic NAC part (residues 61-95) was showed in green region; and the C-terminus (residues 96-140) was showed in red region.

**Figure S8:**

**
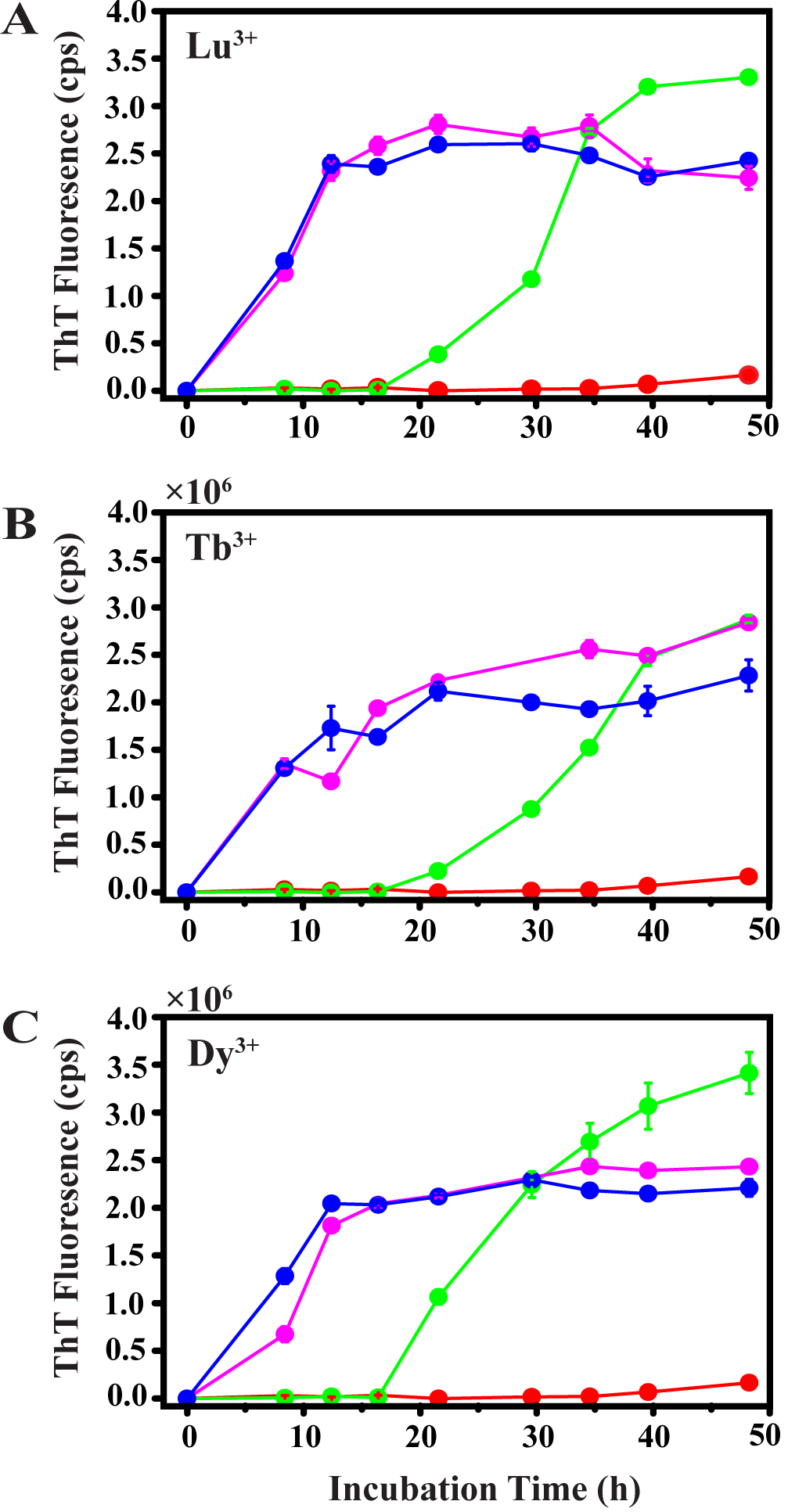
**

**Figure S8:: Fibrillation of α-synuclein monitored by ThT fluorescence in the presence of different lanthanide metal ions**, (A) Lu^3+^, (B) Tb^3+^, (C) Dy^3+^, respectively. 50 μM αS was mixed with 0 μM (red line), 50 μM (green), 100 μM (pink), 200 μM (blue) lanthanide metal ions, respectively. The error bars in figure were standard deviations of three measurements. Points with no visible error bars represent measurements with tiny variance.
